# Children as next of kin’s experiences, practices, and voice in everyday life: a systematic review of studies with Norwegian data (2010–2022)

**DOI:** 10.1177/14034948241232040

**Published:** 2024-03-20

**Authors:** Borgunn Ytterhus, Marit Hafting, Vibecke Ulvær Vallesverd, Eli Marie Wiig, Ellen Katrine Kallander, Marianne Vibeke Trondsen

**Affiliations:** 1 Department of Public Health and Nursing, Norwegian University of Science and Technology, Trondheim, Norway; 2 Child and Adolescent Psychiatrist, Retired; 3 BarnsBeste (Children’s Best Interests) – National Competence Network for Children as Next of Kin, Sørlandet Hospital Trust, Kristiansand, Norway; 4 KORUS Sør (Resource Centre for Addiction, southern region), Skien, Norway; 5 Faculty of Social Studies, Vid Specialized University, Oslo, Norway; 6 Norwegian Centre for E-health Research, Tromsø, Norway

**Keywords:** Children next of kin, children’s everyday life, children’s self-reported health and welfare, young carer

## Abstract

**Aims:** This systematic review aims to identify and describe how children of parents with mental illness, substance dependence, or severe physical illness/injury, experience and practise their everyday life. **Methods:** The review followed the four stepwise recommendations of Harden and colleagues when including quantitative and qualitative studies on peoples’ experiences and views. In all, 23 studies with data from Norway (2010–2022) have been included. Brown and Clark’s thematic analysis was applied. **Results:** Three themes were constructed from the reviewed articles: (a) Children practice their relational agency by actively doing practical tasks, occasionally jobs to maintain family economy, and organising fun activities with the ill parent. (b) Emotional ambivalence when their own needs were set aside in favour of the parents. They loved their parents but also felt guilt, anger, disappointment, shame, fear of inheriting the illness and longed for a ‘normal’ everyday life. (c) Supportive contextual factors were, for example, at least one significant adult recognising them, participating in leisure activities, socialising with friends, and talking with other peers who shared similar experiences as next of kin. Obstructive factors were lack of information and recognition as well as silence and lack of dialogue within the family and/or health professional. **Conclusions:** **There is a strong need for more knowledge and competence on the situation and needs of these children when it comes to professionals, parents and the public. Public health initiatives are needed to honour their agency and recognise their contributions in present time to prevent psychosocial problems later in life.**

## Introduction

The purpose of this systematic review is to identify and describe how children having a parent with mental illness (PMI), parent with substance dependence (PSD) or parent with severe physical illness/injury (PSPI) talk about their experiences and practices in everyday life. We report from studies in which children as next of kin are the participants because we want to raise the children as next of kin’s own ‘voices’. ‘Parent’ includes biological parent, step parent, foster parent and adoptive parent. By children’s ‘everyday life’ we refer to the daily organisation of practicalities and activities as well as the daily experiences that become present in their life world [1]. Consequently, children’s perspectives and agency come first in our review, while research on the healthcare providers’ perspectives and intervention programmes will be omitted.

Having a PMI/PSD/PSPI puts children in the role as next of kin. The term ‘next of kin’ refers to close family membership but differs in different societies regarding inheritance. Parents are close family members. In the Nordic countries the term ‘next of kin’ is used in legislation and public directives as a broad term on children and young people (0–18 years) of a parent being a patient with mental illness, substance dependency or severe physical illness/injury. In our review the concept of next of kin includes ‘young carer’ (YC) and ‘young adult carer’ (YAC), but also those children who adapt to their family situation and use their agency the best they can without being able to articulate verbally their extraordinary childhood and their informal caring. The YC concept was according to Joseph et al. [2] introduced by Aldridge and Becker in 1993 on children and youth under 18 years providing regular, significant and substantial care to ill or disabled family members [2]. In 2008 Becker and Becker added the concept YAC to those young people aged 18–24 years who provide the same [2], while Eurocarers (the European Association Working for Carers) uses the Norwegian Health Directorate’s definition that define YCs up to the age of 26 years [3]. To make sure we did not leave anyone behind, the term ‘next of kin’ includes YCs, YACs, and all children and young people (0–26 years) having a parent during childhood with mental illness, substance dependence or severe physical illness/injury during their childhood or part of their childhood.

Haugland et al. [4] describe children as next of kin to be a diverse and heterogenous group differing in age, gender, developmental and socioeconomic status and living in different family structures. Their common characteristic is that during their upbringing their parents, for shorter or longer periods of time, have had serious illnesses and/or substance dependence. However, there is a huge amount of research documenting that children next of kin having a care role that exceeds their capacity experience a negative impact on their own health and wellbeing [5, 6], and on education and employment prospects [2]. The implications of being next of kin put these children at risk of developing psychosocial problems [7, 8].

The exact number of children who are next of kin is hard to determine [3, 7, 9–12]. In Norway, Torvik and Rognmo [9] have estimated that 23.1% of children are living with a parent diagnosed with moderate or severe mental illness, and 6.5% of children are living with a parent with alcohol dependence to an extent that affects their parental functioning [9]. The Norwegian Health Directorate [3] reports that 18% of Norwegian youth (16–25 years) tell that they have a caring role. In Denmark a register study by Jørgensen et al. [10] identified that 25.3% of 0–21-year-olds had a parent with a severe physical or mental diagnosis. The global prevalence of parents having a chronic medical diagnosis affecting their parental role is estimated at 4–12% [7]. Hanson et al. [11] state that the European prevalence of adolescents carrying out a substantial amount of caring is 7–8%. These numbers correspond with Becker’s [12] estimated number of children under 18 years providing significant, substantial, or regular care as YCs in the UK, Australia, US and sub-Saharan Africa. Even if there are incomparable ways of counting across nations, these percentages illustrate that growing up as next of kin affects large numbers of children nationally and globally.

### The Norwegian context

Until the turn of the last century, research on children as next of kin in Norway has been described as almost non-existent, fragmented and dependent on individuals’ initiatives [4, 13, 14]. Practitioners call for attention to children of PMI/PSD/PSPI. Norway’s ratification of the United Nations Convention on the Right of the Child (UNCRC) [15] led to competence raising through a national competence network for children as next of kin, named BarnsBeste (Children’s Best) in 2007 [16], and in 2010 the Health Personnel Act (HPA) [17, § 10a] and the Specialist Health Service Act (SHSA) [18, § 3–7a] were amended. The first statutory provision deals with minor children as next of kin’s needs for information and necessary follow-up from health professionals on the parents’ consent, and the latter directs all institutions in the specialist health services to have healthcare professionals who are responsible for promoting and coordinating the follow-up of minors of PMI/PSD/PSPI or injured patients. Norway, Sweden and Finland were the first countries to legalise professionals’ duties when parents are ill or substance dependent, which make each of these countries interesting as cases in knowledge production on the topic of next of kin. A few other countries have since followed – for example, Australia (2014) and Iceland (2019).

### Why a systematic review about children as next of kin?

For years the research on children living with PMI/PSD/PSPI has been rooted in a risk/resilience discourse [19]. Childhood has been seen more like a preparatory phase, a state of becoming, rather than a participatory phase in life, a state of being, as emphasised in the new sociology of childhood [20–22]. A focus on the future is still necessary, but it must be balanced by the present wellbeing of children and child participation. Consequently, their voices need to be heard in all matters affecting them [15] within different contexts during their childhood [15, 23].

We have identified eight international reviews published after 2010 examining experiences of everyday life of children with PMI/PSD/PSPI [24–31]. Three of these reviews only examined qualitative studies on children’s experiences of a parent with mental illness [24, 27, 28]. Two examined the consequences for children with a physically ill parent [25, 26], and two focused on YCs [29, 30]. Steffenak et al. [30] was the only review that focused on community-based support across parental illnesses. What did their results tell?

Chikhradze et al. [29] found that even though YCs were highly involved in providing care they did not want to be identified as a YC, but rather as an ordinary child. Their finding contrasted with that of Saragosa et al. [31] in which YCs accepted their role and wanted to relate to others with similar experiences. In addition, Saragosa et al. [31] examined the children’s interaction with the healthcare system and recommended creating training tools to help professionals identify young carers.

Gladstone et al. [24] reported that children wanted to keep the family together and worried about separation from parents during hospitalisation. Children talked about love and the good days they had with their parents, but also their experiences of a range of difficult emotions associated with stigma, shame, embarrassment, guilt and fear. While Gladstone et al. [24] called for more knowledge on children’s everyday life experiences and evaluations of implemented intervention programmes, Dam and Hall [27] argued that breaking family concealment with information, dialogue and knowledge seems to be the best remedy for children. Yamamoto and Keogh [28] questioned if we understand what children of parents with mental distress really need, which is a prerequisite for providing relevant services.

Järkestig-Berggren and Hanson [26] concluded that few of their reviewed studies focused on external outcomes – for example, school improvement, social networks and resilience. In addition, they found a lack of information about parents’ and children’s experiences of the effectiveness of interventions. They confirmed the importance of having a significant adult in children’s lives when a parent is ill, and underlined the need for more knowledge about preschoolers as next of kin. Krattenmacher et al. [25] reviewed quantitative studies on associative factors with children’s psychosocial adjustment in nearly 300 families with a parent with cancer. Their findings showed that better family functioning indicated better child adjustment, whereas a parent’s depressive mood indicated worse adjustment for the children. In addition, Krattenmacher et al. [25] report that the parents’ health status and medical diagnoses had no direct impact on children’s adjustment, while the samples in five of the above-mentioned reviews [24–28] were based on the parents’ medical diagnoses.

One review asked for more knowledge on how age and gender influence children’s experiences as next of kin [25]. Another review asked for more knowledge on gender and on migrant children’s experiences as next of kin [29]. Two reviews underlined children’s need to be recognised as next of kin [30, 31], while one underlined the importance of children receiving support according to their preferences, eliciting children’s voices and ensuring that community health and social services are developed for and ‘tailored’ to children [30]. Confidentiality and anonymity were found to be important for the children. Some children also had ambivalent feelings about the healthcare services [29, 31].

When summarising we can say that these eight reviews provide a broad knowledge base for understanding children's everyday life, like, however, also some important shortcomings in current understanding and care. A more and deeper understanding of the discrepancies in the children’s self-image was asked for [29, 31]. In addition, more knowledge is needed on children’s voices in all matters affecting them [30] to ensure the development of programmes adapted for their preferences. Differentiated knowledge on how age, gender and immigrant background influence the role as next of kin is also questioned [25, 29], as well as on external outcomes, for example, school improvement, social networks, resilience and children’s experiences of interventions [26]. Studies including Norwegian data are almost non-existent in the above-mentioned reviews.

Based on the knowledge from the eight existing reviews and what they found missing we became curious about what research including Norwegian data could contribute with. In our present review we have chosen to elicit how children’s voices, acting and experiences in everyday life come into being across the parents’ medical diagnoses. This context led us to the following research question:

- What do we currently know about children as next of kin’s experiences, practices and voices in everyday life in Norway (2010–2022)?

## Methods

This is a systematic review following the four stepwise recommendations of Harden et al. [32] when including quantitative and qualitative studies on peoples’ experiences and views. First, they recommend that we make a clear research question based on earlier reviews and contextual information. Second, we clarified the sample through a thorough systematic categorisation of the full-text articles according to outcome focus and exclusion criteria as reported in line with the Preferred Reporting Items for Systemic Reviews and Meta-Analyses (PRISMA) 2020 in the flow diagram. Third, a quality assessment is needed on the included studies according to the recommended seven criteria of Harden et al [32]. Fourth, the final step was to do an extraction of the findings from the studies using a thematic content analysis [32].

### Sample and identification of articles

**Figure 1.**
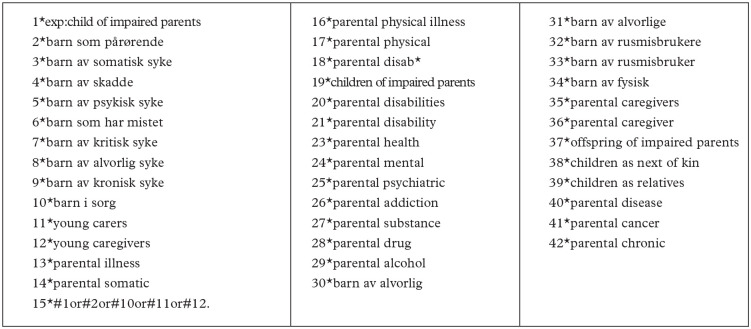
The search term used when the professional librarian looked for scientific reviewed articles in Scopus, MedLine, Norart, Swemed and Oria. Search has been carried out with different combinations of AND, OR and NOT.

The sampling procedure started with a broad systematic search in library databases together with an academic librarian at the Norwegian University of Science and Technology (NTNU). Searches were performed in Scopus, MedLine, SweMed, Oria and Norart from 1 January 2010 to 1 April 2022, for articles written in English or Norwegian. The broad search was made to catch as many aspects as possible of children as next of kin’s situation, their ill and dependent parents as parents and the professionals’ contact with these families. The search terms are reported in Figure 1.

**Figure 2.**
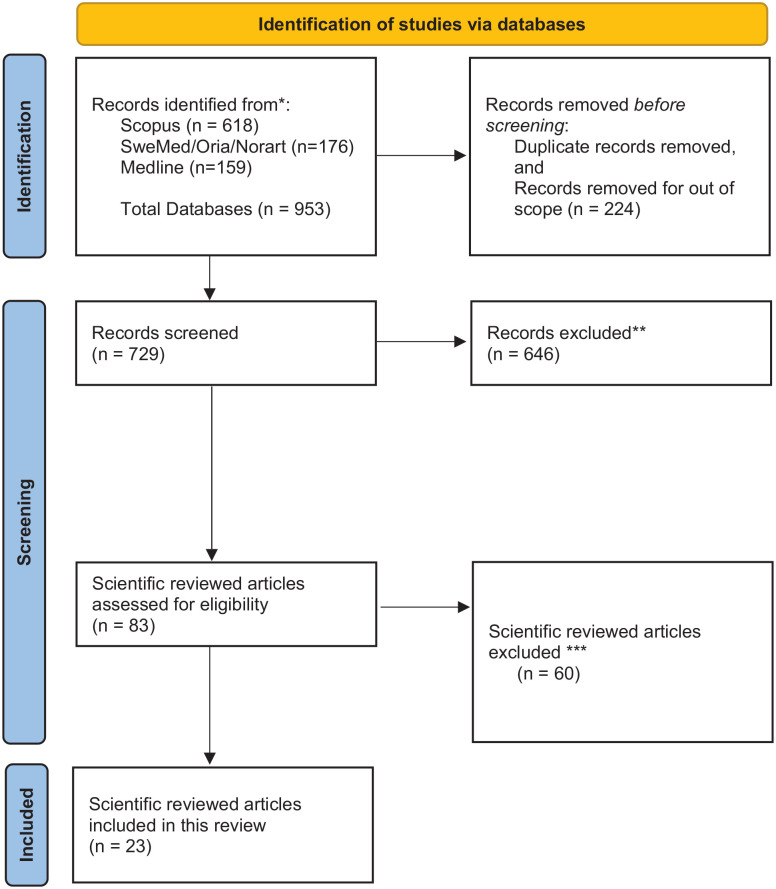
Flowchart of search and selection of articles. **The exclusion criteria: studies in languages other than English or Norwegian; studies without data from Norway; studies in which the child suffered from illness or had an impairment; studies that only focus on experiences of service providers and/or the private social network of the ill parent; studies on children with deaf or hard-of-hearing parents; studies on children of parents with intellectual disabilities; studies on adult children with old parents; studies on unborn children/fetuses of ill or substance-dependent mothers; articles published as chronicles, commentaries or editorials, review analysis; articles that have not been through scientific peer review processes; book chapters; protocols and grey reports. ***All records were excluded by the authors due to focus on the ill or substance dependent parent/spouse and healthcare professionals and only indirectly on the child as next of kin, see the procedure described in the text. Articles without focus on the children’s experiences, practices and voice in everyday life during childhood were excluded.

The keywords were combined in different ways in each language. The sampling procedure was carried out in close collaboration with a professional academic at the first author’s university library. Through the PRISMA identification and sampling procedure we identified 953 studies (Figure 2). The screening procedure was carried out manually. We went through the reference lists of the former review articles introduced in the introduction part of our article. However, no new studies based on Norwegian data and published after 2010 were identified and supplied to the systematic search guided by the librarian. All six authors reviewed the identified abstracts, and the decision to include/exclude abstracts was reviewed jointly. If we were unsure about an abstract, it was reviewed once again by two authors before making the final decision. We used the following exclusion criteria when records were screened: studies in languages other than English or Norwegian; studies without data from Norway; studies in which the child suffered from illness or had an impairment; studies that only focus on experiences of service providers and/or the private social network of the ill parent; studies on children with deaf or hard-of-hearing parents; studies on children of parents with intellectual disabilities; studies on adult children with old parents; studies on unborn children/fetuses of ill or substance-dependent mothers. In addition, we excluded articles published as chronicles, commentaries or editorials, review analysis, articles that have not been through scientific peer review processes, book chapters, protocols, and grey reports. When reviewing articles assessed for eligibility all records were excluded by the authors due to focus on the ill or substance-dependent parent/spouse and healthcare professionals and only indirectly on the child as next of kin. To sum up, only articles with focus on the children’s experiences, practices, and voice in everyday life during childhood were included.

We ended up with 83 articles for full-text reading and thoroughly and systematically reviewed approximately an equal number of the-full-text articles. Articles that were hard to determine were re-read by at least two authors. Twenty-three studies (26.4%) in the included sample examined children as next of kin’s experiences of everyday life when living with a PMI/PSD/PSPI, and we then proceeded to review them systematically. The flow diagram in Figure 2 illustrates the sampling and screening and the exclusion criteria.

### Analyses

Three of the authors quality assured the 23 articles according to the seven quality criteria of Harden et al. [32]. The result of the assessment is presented in Table I.

**Table I.** Number and percentage of studies (*N*=23) satisfying each quality criterion.

Criteria	Number	%
1. An explicit theoretical framework	14	60%
2. Aims and objectives clearly stated	23	100%
3. A clear description of context	23	100%
4. A clear description of the sample and how it was recruited	23	100%
5. A clear description of methods used to collect and analyse data	23	100%
6. Attempts made to establish the reliability or validity of data analysis	23	100%
7 . Inclusion of sufficient original data to mediate between evidence and interpretation	23	100%

The criteria were discussed by the authors who assessed them as equally as possible. All the included studies scored on criteria 2–7. However, 14 of the 23 (60.1%) also scored on criterion 1: ‘An explicit theoretical framework’ [34, 35, 38, 42, 46, 47, 49–56]. The theoretical framework is here the logically developed and connected set of premises and concepts the researchers use as a lens when developing data/grounding their study. In line with Harden et al. [32] we carried out their recommended quality assessment tool for systematic reviews including both qualitative and quantitative studies. Studies including a conceptual framework which here means they included the empirical state of the art, knowledge gaps and methodological underpinnings are defined as not having an explicit theoretical framework. A reduced score on only one criterion did not qualify to weigh these studies’ contributions in the presentation of findings or in the discussion. The thematic analysis [33] of the 23 articles was carried out, and the identified themes and findings are presented in Table II [34–56].

**Table II.** Children’s reported experiences in everyday life 2010–2022 (1 April).

Results: What it is like in everyday life to be a child as next of kin						
Authors. Year. [number of full references]	Participants	Data source	Outcome measures	Emotionally	Contextually	Children’s agency
**Bønnhoff & Larsen, 2014 [34]**	Texts from authors who have grown up with parents with alcohol abuse	Four published self-biographical novels, autobiography	How their relations with their alcohol-dependent parents have shaped them	Dilemmas in disclosing the difficulties in the family Felt shame, guilt, stigmatisation and ambivalence	Parents’ needs always came first The importance of maintaining the family façade; parents’ self-delusion	The use of dialogue and reflections to separate themselves from the parent Moved out from their family home Searching for the parent’s ‘true’ face behind the alcohol intoxication and the facade
**Eide et al., 2020 [35]**	Adolescents (13–18) with severe somatically ill parents	11 In-depth interviews	Adolescents’ experiences as next of kin	‘Living in an earthquake zone’ Mind occupied with troubled thoughts and feelings about the parental illness	Increased family closeness Experienced challenges in their relationship with friends Ethical dilemmas concerning whose needs to cover when	Experienced personal development based on their role as next of kin
**Faugli et al., 2021 [36]**	Children (8–18) with parents having physical illness, mental illness or substance dependence	238 Open-ended texts from questionnaire	Describes what children with parents with physical illness, mental illness or substance dependence experience as most difficult	Troubled thoughts and feelings, which strained their cognitive and functional levels Fear of parent dying	Challenged relationships with friends Negatively impaired relationship with their parents, friends and others	
**Gullbrå F et al., 2016 [37]**	Adolescents (16–25) with parents having physical illness, mental illness or substance dependence	Three focus group interviews	What GPs should identify and recognise to improve the adolescents’ everyday life	Unpredictable everyday life	Feel that GPs are not pro-active according to their family situation Want information and support from GPs	
**Hansen & Ersfjord, 2021 [38]**	Children (5–8) with parents with type 1 diabetes (T1D)	Brainstorming Conversation included: Associative exercise Body map and diabetes supplies Diet exercise Semi-structured individual interviews	Explore the children’s management, experiences, understandings of T1D	Children worried about their parent’s current health and later in life Feared not being able to help the ill parent without an extra adult present Fear of parent dying	Differs much according to what parents say and the child’s curiosity and maturity	Know the emergency-call number Gives the parent chocolate milk if the monitor ‘beeps’
**Haugland et al., 2020 [39]**	Young people (18–25) with parents having physical illness, mental illness or substance dependence or disabilities	Sample from a national survey (SHoT2018) *N*=2220	Care responsibilities Mental health Somatic health Life satisfaction	Number of hours providing care was associated with negative outcomes in a close pattern	Care responsibilities were associated with: girls/young women, singles, having divorced parents, being an immigrant, having financial difficulties	Young people who are female, single, have an immigrant background, or divorced parents take on more care responsibilities
**Haugland et al., 2022 [40]**	Young people (18–25) with parents having physical illness, mental illness or substance dependence or disabilities	Sample from a national survey (SHoT2018) *N*=2228	Study progress Number of close friends Loneliness Psychical exercise Involvement in organised volunteer student activities	Lonelier than students without care responsibilities	More likely to report delay in study progress Higher number of failed exams Slightly fewer friends Those providing more than 2 h care per day participated less in leisure activities	More likely to participate in cultural activities and student democracy
**Jeppesen et al., 2016 [41]**	45 Families with one parent with cancer and their 69 teenagers	HRQoL assessment KIDSCREEN-27 compared with European Normative Sample NORMs	HRQoL Gender differences	The teenagers scored lower on physical wellbeing compared with NORMs Girls scored lower on HRQoL than boys Teenagers’ self-esteem; influence on teenagers’ HRQoL	Family and parental cancer related characteristics did not influence teenagers’ HRQoL	
**Johannesen et al., 2016 [42]**	Young people (18–30) with parent with dementia who were younger than 18 at the onset of dementia	14 Qualitative depth interviews, twice Longitudinal design	The young ones’ experiences of dementia in their everyday life	Influenced their wellbeing negatively Anger, sadness, guilt Everyday life gets better one year after dementia onset	Important to have social support Important to be seen and listened to Get response to their needs	Humour as a strategy to handle sadness Moving away from home Greater personal distance Calmer emotional reactions, which made detachment easier
**Kallander et al., 2018 [43]**	Children (8–17) with parents having physical illness, mental illness or substance dependence in specialised health care	246 Children carried out multidimensional assessment of care activities. (MACA-YC 18)	Extent and nature of care activities and whether these are associated with family function, demographics, children’s characteristics Differences between care activities and parent’s types of illness	Six in 10 reported emotional care in line with children in general population. High levels of emotional care are associated with high extent of social skills	Only 6% of the parents received home-based service 25% of children with parents having mental illness or substance dependence report more than 10 h of care activities a week, only 4% of those having parents with physical illness, but they did more domestic care than the other two groups Poor ADL functioning in parents, independent of illness increased the extent of care activities and household tasks	Being a girl, higher age, lack of control and better social skills were associated with higher extent of caring activities
**Kallander et al., 2018 [44]**	Children (8–18) with parent having physical illness, mental illness or substance dependence in specialised health care	238 Children Multicentre study PANOC score	The positive and negative outcomes for children who care for an ill or dependent parent Differences in positive and negative outcomes of care activities across the three parent groups Whether children’s outcome from caregiving is associated with family functioning, family demographics, children’s characteristics	10% Reported negative outcomes of care activities at a clinical level of concern (life not worth living, sadness they could not handle) Children with substance-dependent parents had more negative outcomes than the others 90% reported it was positive for them to contribute, although 50% reported stress No significant differences across parental groups	Fewer personal care needs, fewer financial problems, practical and household management predict more positive outcome	Better social skills were associated with more positive outcome Higher levels of personal care for the parents, more financial and practical management were predictors of more negative outcomes
**Kallander et al., 2021 [45]**	Children (8–18) with parents having physical illness, mental illness or substance dependence in specialised health care	246 Children carried out health related quality of life (HRQoL) KIDSCREEN-27	Factors associated with self-reported HRQoL	QoL negatively associated with children’s provision of emotional care for the ill parent and self-reported responsibilities due to the parental illness	The QoL increased when other adults took over the responsibility for the ill parent and sibling care	Children’s self-reported QoL was positively associated with their self-reported social skills
**Martinsen et al., 2019 [46]**	Young people (14–22) with parents with mental illness	Seven individual semi-structured interviews	Young people’s need for involvement and information Coping strategies when coercion is involved	Ambivalence Guilt when taking the initiative for coercive action towards the parent Afraid Felt need for interconnections and preservation of relationship with their ill parent	Lack of information from healthcare professionals Lack of interaction with the ill parent when hospitalised	Struggling to keep up the relationship with the ill parent Take an active role in keeping the family together
**Mauseth & Hjälmhult, 2016 [47]**	Adolescents (12–18) with a parent with multiple sclerosis (MS)	15 Individual semi-structured interviews	Identify the adolescents’ concerns in daily life How adolescents cope with these concerns	Preserving control in an uncertain everyday life Difficulties in balancing their own needs with their parents’ needs Fear of inheriting illness/disorder	Lack of information from healthcare professionals and family Lack of professional support Financial challenges Had to do many chores	Take on many responsibilities in balancing needs Reflecting Adjusting Seeking respite Shared difficulties with significant adults Did not initiate information on internet themselves
**Torp et al., 2013 [48]**	386 Cancer patients living with children <18 answered a questionnaire 10 children <18 with a parent with cancer	10 Individual in-depth interviews	Describes the children’s experiences regarding the impact of cancer on their socioeconomic situation	Simply being with their friends and maintaining a ‘normal’ life Afraid their ill parent could die Those 10–13 had difficulties concentrating at school	Children reported that dialogue with health professionals about their parent’s situation was important Generally, not very concerned with the financial situation	Do something fun Reluctant to ask parents for money, taking on a part-time job
**Trondsen & Tjora, 2014 [49]**	Adolescents (15–18) with a parent with mental illness	13Iin-depth interviews with adolescents participating in an online self-help group	If and how the online self-help group may provide support for the adolescents in everyday life	Shared worries about their own reactions and feelings according to normality Shared feelings of loneliness, loss and sorrow Fear of inheriting illness/disorder	Left alone with the impact of their parents’ illness Silence, lack of openness Lack of information The online self-help group was the first place to share feelings and thoughts for some of the participants	Retaining independent active steps towards plans and ambitions Normalised each other Discussed secret experiences and feelings Recognised each other’s experiences Strategies for managing everyday life
**Trondsen, 2012 [50]**	Adolescents (15–18) with a parent with mental illness	Participant observation in an online self-help group for adolescents with parents with mental illness	Adolescents’ experiences of everyday life when living with a parent with mental illness	The adolescents felt loneliness, loss and sorrow Some were afraid of dying; that their parent could be suicidal or attempt suicide again	Their everyday life was unpredictable and unstable Lack of information inside and outside the family	Taking responsibility for household issues, caring for siblings and the ill parent Constantly switching ‘emergency alert mode’ on Taking time out to create physical and emotional distance from their parents Seeking professional support Moving away from home
**Wangensteen et al., 2019 [51]**	Young people (13–26) with parents with substance dependence	12 Individual qualitative interviews	The young people’s perceptions and reflections of living with a parent with substance dependence	Mixed and contradictory emotions towards parents Insecurity Afraid and fear of parent dying Anger and disappointment Grief and sadness Love and felt sorry for the parent	Lack of professional support and trusted adult they can share their experiences and feelings with	Tries to stand up for the parent when everybody else has let them down Ask for professional support Attempts to determine regulation and type of contact with the parent
**Wangensteen et al., 2020 [52]**	Young people (13–26) with parents with substance dependence	7 Individual qualitative interviews	The participants’ experiences of their parents’ substance dependence in their everyday lives	Felt different from others Blaming themselves Felt unworthy and lonely	Incomprehensible behaviour that was hard to understand Wanted more information about substance use problems Being bullied, stigmatised and prejudiced against	Tried to understand the development and maintenance of their parent’s substance dependence Tried to hide the substance dependence from family and friends
**Wangensteen & Westby, 2021 [53]**	Young people (21–26) with parents with substance dependence	Five in-depth interviews	The young people’s reporting of protection factors	Awareness of own vulnerability /predisposition to substance dependencies Being afraid, worried and insecure during childhood Felt lonely and different during childhood	Important safe living conditions: The child welfare services Move to a foster home Having one significant adult Some talked about respectful and caring conversations with professionals	
**Werner & Malterud, 2016 [54]**	Adults who grew up with parents with alcohol dependencies	Nine individual semi-structured interviews Retrospective data on everyday life issues in childhood	Explore results of encounters with professionals in childhood with respect to parents with alcohol use and their everyday life	Felt ignored and let down by professionals Felt threatened and ashamed Concentration- problems at school	Believed that professionals rarely recognised the family’s problems Children’s struggle was probably noticed, but adequate response never came Professionals working with the parent seem to avoid further involvement	
**Werner & Malterud, 2016 [55]**	Adults who grew up with parents with alcohol dependencies	Nine individual semi-structured interviews Retrospective data on everyday life issues in childhood	Children’s role and experiences in childhood	Deafening silence Felt betrayed by the other parent who trivialised the alcohol-dependent parent Felt uncertain and insecure	Lived in an everyday drama characterised by tensions, threats, manipulation and blame Family conflicts Very tense atmosphere in the family Disturbed family activities, routines and celebrations Concealing the difficulties, and taboo to talk about them outside the home Limited family and professional support	Tried hard to manage and restore social order in the family Performed loyally to maintain normality Concealed the problems Avoided bringing friends home Played the role as family mediator
**Werner & Malterud, 2017 [56]**	Adults who grew up with parents with alcohol dependencies	Nine individual semi-structured interviews Retrospective data on everyday life issues in childhood	Explore informal adult support experienced in childhood	Explicit recognition from adults made children safe Implicit recognition led to ambiguity	The importance of participating in activities relevant to their age without being confronted with their role as next of kin One significant trusty adult representing ‘a safe harbour’ providing a sense of normality	

## Results

We realised that 11 [43–45, 49–56] of the 23 included articles in the present review build on data from four studies, which illustrates that in the data from 12 years of research on children’s experiences our review has been able to identify 23 articles, but they built on only 16 different studies. Consequently, children as next of kin’s voices are heard in fewer Norwegian studies than the number of publications indicated.

In most of the 23 included articles they referred to the age-span of the children as 8 to 26 years, half between 8 and 19 and half between 19 and 26 years. However, five articles stood out: in one of them the 12 participants were between 5 and 8 years [38]. In three studies the nine participants were adults up to 54 years of age who recalled their childhood with substance-dependent parents [54–56]. The data in Bønnhoff and Larsen's analysis [34] are autobiographical texts about childhood experiences and age is not included. In total, this means that the bulk of the material in this review is from adolescents and young adults, and children younger than 5 years are involuntarily absent in the sample.

Based on the described systematic review process three themes are constructed based on what pervaded the articles when it came to the children’s everyday life experiences and practices: (a) emotional ambivalence: promotive and hampering factors for wellbeing; (b) supportive and obstructive contextual dimensions of wellbeing; (c) children’s relational agency helped them create meaning. These three main themes will be presented separately.

### Emotional ambivalence: promotive and hampering factors for wellbeing

The first main theme refers to children’s experiences of emotional ambivalence when having PMI/PSD/PSPI, which means they experienced contradictory emotions towards the ill parent and the entire family situation. On the one hand, they loved and cared for their ill parents, but at the same time struggled with such feelings as guilt, anger, disappointment, shame and longing for a ‘normal’ family life. Children expressed how they oscillated between hope and hopelessness, love and hate with parents with substance dependence [34, 52].

In the following we will first present what strengthened the children’s positive emotions and wellbeing, and then present what moved the children’s wellbeing in a negative direction.

#### Maintaining wellbeing and a ‘normal’ everyday rhythm

In a study on children of parents with multiple sclerosis (MS) some of the children felt that their everyday life with an impaired parent had made them more responsible, independent, patient and empathetic than their peers [47]. Other children expressed pride because they had been able to break the cycle of substance dependence problems in their family [52]. Some of the studies found that the ability to maintain daily routines was important for the children with PMI/PSD/PSPI. Based on children’s self-reported data from KIDSCREEN-27, Kallander et al. [45] found that the children’s self-reported quality of life (QoL) score was positively associated with the ill parent’s self-reported physical health, and that other adults took over the parent’s responsibilities and care provision of siblings and the ill parent. In addition, health-related QoL was positively associated with the child’s own social skills [45] and with being a boy [41, 45]. The ill parents’ medical diagnoses had a moderate effect on teenagers’ reported QoL [41].

#### Difficult emotions regardless of their parental health problems

Difficult emotions were expressed by the children regardless of parental health problems. Many of the young people said they felt lonely because no one talked to them about their parents’ health problems and illnesses and the consequences for their daily life [46, 49, 50]. Others expressed that they felt betrayed by the other parent who might trivialise the alcohol problems of their partner in the family situation [55]. Lack of information and openness gave a sense of being ‘invisible’ in the family, and a feeling of being the only one in the world experiencing a mentally ill parent [50]. The feeling of loneliness also made young people uncertain and insecure [52, 55]. Some of the youths with a mentally ill parent expressed that the silence and failure to talk about the problem was a source of concern, fear and frustration [49]. One of the informants in Martinsen et al. [46] said that lack of openness about the mentally ill parent can be a significant challenge in family life:

What has been the greatest problem in our family is that we haven’t talked about it at all. Although it has had such a strong impact. . . . Instead, we have tried to hide from each other, and in a way, run away from it. [46, p. 217]

Children expressed that such deafening silence made them feel let down by adults in the family and by professionals, whom they felt should have noticed what was happening in their family [34, 49, 50, 55]. Moreover, children and youth in many of the studies said they felt shame, guilt and stigmatisation over their parent’s illness or substance dependence [34, 46, 47, 49, 50], and consequently disguised their parent’s problems. In a study by Bønnhoff and Larsen [34], the participants’ stories illustrate the difficulties in disclosing the parent’s alcohol problems, even though its consequences dominated the whole family’s everyday life.

On standardised questionnaires 50% of the participating children as next of kin reported stress. The more hours spent on providing care, the more stress they reported [44]. In another article [43] children did not report significant differences in the extent of the provided care between their parents’ types of illnesses, but there were differences in the types of activities, and more than one in 10 reported providing care for more than 10 h a week on average. In Kallander et al. [44], children with mentally ill parents reported limited wellbeing more often than children with parents with a physical illness or substance dependence. However, children with parents with substance dependence reported psychosocial problems on a level of concern more often compared with those with parents with physical or mental illness. Wellbeing and psychosocial problems were associated with the nature of care activities – for example, personal care, household, financial and practical management [44]. However, in Jeppesen et al. [41] youths who had parents with cancer reported having a lower physical activity level, being more easily physically exhausted, feeling physically unwell and having a lower energy level.

Unpredictability in childhood relations hampered children’s wellbeing. Adolescents of parents with neurological disorders or cancer reported that they felt as if they were ‘living in an earthquake zone’ because their parents’ illness appeared out of nowhere [35, p. 5], and one of the adolescents described it as: ‘It’s like a bang, kind of, getting sick. Because it happened so suddenly’ [35, p. 5]. Similar findings are reported by Trondsen and Tjora [49] in a qualitative study of an online self-help group for adolescents with mentally ill parents. The participants in the study mentioned the unpredictability and instability of daily life following the mental illness as being particularly challenging and overwhelming. One girl said: ‘My mom has rapidly fluctuating depression. She has one completely normal day, and on the next she doesn’t want to talk to anyone and just cries and rants.’ [50, p. 179].

Children reported guilt from two different angles: because of active involvement in the parents’ care and treatment, and due to avoidance of such involvement. For example, some of the young people with mentally ill parents said they felt guilty because they had taken the initiative to initiate coercive treatment for their parent [46]. Several of the informants in Martinsen et al. [46] used such words as ‘hard’ and ‘tough’, stating they often felt upset, depressed, irritable, angry, introverted and silent in their own everyday life with a mentally ill parent. Gullbrå et al. [37] found that avoidance of initiating coercive treatment was reported as the source of guilt. Children as next of kin often felt guilty because they had done too little to support their parents [37, 46], reporting anger or frustration. One girl (aged 18 years) said:

All my life I have been working with my feelings of guilt. And it’s all about my mum. I have to deal with it when she is down. Then I am thinking – it is my fault. And she can say: ‘Yes, it is your fault.’ And suddenly she says: ‘No, it is my fault!’ It is very hard to cope with her inconsistent mood.” [37, p. 363]

Such feelings were mentioned by children regardless of their parents’ medical diagnoses.

#### Fear of inheriting the illness and death

The children expressed concerns of inheriting their parents’ medical diagnosis, as seen in several studies on mentally ill parents [46, 49], and parents with MS [47] and substance use disorders [53]. In Wangensteen and Westby [53, p. 160] one informant said: ‘I drink alcohol, but I certainly have no substance use problems. I have been afraid of developing substance use disorder.’ Trondsen and Tjora [49] found that the adolescents participating in their study knew very well that they represented an at-risk group for developing mental illness, and in situations where they felt especially sad, they feared they were developing depression themselves.

Several of the reviewed studies [36, 38, 49, 50] reported that the children and youths expressed the fear that their parent would die because of the illness, and the fear of suicide, overdose or an accident. In the only study of preschool-age and early primary school aged children [38], an 8-year-old boy with a father who had type 1 diabetes expressed: ‘I get a little bit sad because he (his father) can die’ [38, p. 717]. This young boy also reported practical worries related to this issue. He was afraid of being alone with his dad if no other adult were present because he knew he could fall due to his irregular blood-sugar level: ‘. . .what if mom does not know about it? (I am) not able to lift daddy up. . . .’ [38, p. 717]. Not being able to support the parent with cancer or neurological disorders was a concern for some children [36].

In the study by Trondsen and Tjora [50], the participants with mentally ill parents expressed a deep fear of suicide. They talked about frightening memories of finding suicide notes, searching for their disappeared, suicidal parent and their parent’s hospitalisation after suicide attempts. One girl whose father had made suicide attempts earlier said she was afraid it could happen again: ‘I was scared that I one day would come home and find him dead’ [50, p. 180]. Similar worries were expressed by youths who had a parent with substance dependence, when they feared their parent could die due to an overdose [51]. One young man (aged 21 years) expressed his ambivalent feelings and fear of losing his mother with substance dependence to an overdose:

What is crazy about this is not that she is using drugs, but the fact that everything seems to be so fine when she is in treatment. Then, just a couple of weeks later, it is back to hell again. (. . .) The only thing in life I am afraid of, is that she will die. [51, p. 203]

This illustrates that fear of dying and ambivalent emotions intersect with the specific context.

### Supportive and obstructive contextual dimensions of wellbeing

The second main theme in our review refers to children’s descriptions about the relational, structural and physical contexts as important in everyday life with parental illness [34, 35, 38, 42, 46, 47, 49–51, 53]. We will first present findings that describe what children understood as the supportive nature of the contexts, and then their experiences of obstructive contextual elements.

#### Supportive contexts increase wellbeing

The children and youths described that significant adults, and respectful, caring dialogues with professionals who recognised their life situation made everyday life secure, and kept their daily rhythm stable. Some talked about crucial interventions by the child welfare services – for example, moving from lability and insecurity to a stable foster home or moving out of the parental home [34, 37, 42, 50].

Children in several of the studies talked about the importance of a trustworthy person. This could be the healthy parent, a relative (often grandparents), social worker, teacher, friend or other peers [34, 37, 49, 50, 53]. The support could be of an emotional and/or practical nature. A girl (aged 18 years) who had a mentally ill mother said:

It is so lovely to talk to some adults who can tell you that this is NOT how you should live. You should not do the dishes after a huge dinner that you didn’t eat. That is not how it should be for a kid. You should be out playing because it is sunny outside. That kind of information is so incredibly important. [37, p. 363]

Adolescents of mentally ill parents stated that having one trustworthy and available person was an important strategy for managing their challenging family life [42, 49, 50]. In a study of an anonymous online forum for adolescents with mentally ill parents, Trondsen and Tjora [49] found that the participants shared and exchanged experiences about difficult emotions and challenges, and discussed how to manage their everyday life as next of kin. Understanding that they were not alone in dealing with complex emotional and practical situations gave them hope and made it easier to cope with their life situation [49].

Other children said that the ties between themselves and their ill parent became safer and more bonded after some time with parental chronic illnesses [35, 42]. Hansen and Ersfjord [38] point out that the youngest children (5–8 years) in their study did not seem to worry that much about their parents’ diabetes if they had been told how to behave if the parents’ behaviour changed. Johannesen et al. [42] found in their study of children who had parents with dementia that it was extremely hard for teenagers to lose their parent cognitively. However, they experienced that moving out of their parents’ home early made it easier to live their own life, and then their relationship with their ill parent improved [42].

#### Obstructive contexts create worries and heavy workload

Both quantitative and qualitative studies have found children and youth who said they felt practically and emotionally lonely [36, 40]. Loneliness was especially challenging for children living with a single ill parent. One boy (aged 15 years) said in the study of Faugli et al. [36, p. 5]: ‘The most difficult challenge is that I have to get up early in the morning, all alone, wake up my siblings, and make breakfast and lunch for them, and so on.’ Housework and caring for siblings were reported as work that occupied too much time for some participants [36]. Young adults with care responsibility for ill parents reported that they used more than 2 h a day providing care, and participated less in sports and recreational activities than their peers [40].

In a study by Werner and Malterud [54], adult children who had grown up with parents’ alcohol dependency said the greatest challenge was the drunk parent’s changes in behaviour, such as aggressiveness, saying inappropriate things, and threatening behaviour. Moreover, they found it challenging that the alcohol problems were disclosed within the core family:

If mum was very drunk, and I said ‘Now you are drunk. Would you please go to bed? I cannot deal with this now’ she would stand there, saying ‘oh, you always say I am drinking. I am tired after work.’ [55, p. 5]

The informants experienced that instead of taking responsibility or admitting that they had a problem, the parents with alcohol dependence neglected and blamed their children for being wrong, too strict or lacking empathy. In addition, they felt that the other parent often trivialised the problem and took the side of the parent with substance dependence more often than siding with the child. The informants reported that it seemed like everyone hoped that someone else would raise the alarm, but in the end nobody acted [55]. However, some children described how it was good for them to know that an adult who knew their family situation and recognised their role as next of kin was present in situations when the child participated only in the role as an ordinary child, for example, at leisure activities with peers [56].

Young adults (18–25 years) with care responsibilities for substance-dependent parents reported in a national survey that they were lonelier and had slightly fewer close friends than their peers [40]. In the qualitative study of Trondsen and Tjora [49, p. 180], adolescents of mentally ill parents also expressed loneliness even though they had good friends, because their friends ‘will never understand. I feel very lonely in the middle of this.’ In other studies, some of the informants expressed that they protected themselves from being seen solely as vulnerable, sad and suffering by not telling friends about the situation at home [35, 37]. The study of Gullbrå et al. [37] of GPs working with children as next of kin found that the whole community, including the GP, seemed to neglect the young peoples’ needs. One of their participants said:

My mum is an alcoholic, and when I was young and lived with her, she was almost always drunk. Everybody should have seen that I could not live there. At that time, my mum and I visited our GP together, and the GP should have alerted someone. Because that should be a doctor’s job. [37, p. 364]

These young people often visited their GP for various somatic problems – for example, headache, stomach ache, muscle complaints or anxiety. However, they experienced that the GPs mostly limited their response to these symptoms, and did not ask about their everyday life and distress because of their parent’s illnesses [37]. Some adolescents reported financial consequences for their family due to the parents’ illness, and that occasional jobs helped them to pay for things their peers’ parents financed [47, 48]. For some, financial and practical problems seemed to be almost as stigmatising to talk about outside the core family as the illness itself [47].

### Children’s relational agency helped them create meaning

The third main theme in our review refers to children as relational actors and their expectations of being recognised as such [42, 47, 49, 50, 56]. In the present article agency is understood as:

. . . an ever-present characteristic in every human: The child influences her environment by her actions, by being a body, and by being connected to certain ideas/ideologies, and is as such a co-creator of social structures. Children . . .are competent and agentive as well as vulnerable and dependent on others. [57, p. 49]

The children in the included studies interacted on their own premises and were both influenced by their context and influenced their context. In all the studies that indicated children’s agencies, they mentioned that children took responsibility and tried to maintain their everyday life [39, 42, 47, 50]. In the studies by Mauseth and Hjälmhult [47] and Johannesen et al. [42] the children said that doing practical tasks provided relief from worries and uncertainty, and made them confident by adding meaning to everyday life. Children described practical tasks initiated by themselves as different from tasks imposed by their parents or other family members. Some children reported that they tried to organise fun activities with their ill parent to make the parent feel better, but they refrained from activities if they saw their parent was having a bad day [42, 47]. Informants who had grown up with parents with early dementia mentioned humor as an outlet, and said it gave them emotional relief and helped them to manage difficult situations [42].

Another active strategy was to talk with other children and youths as next of kin to share experiences and information, which could be done digitally in a self-help forum [49, 50] and in physical encounters with peers [42, 52]. In the online self-help group for adolescents with mentally ill parents the participants shared challenging experiences and emotions from their everyday lives, and gave each other advice and support [49]. Communication with peers was experienced as helpful in re-framing hopelessness into hope and taking active steps towards future plans and ambitions [49].

In a qualitative study of children with a parent with diabetes, all the children (5–8 years) expressed important practical knowledge on how to behave in specific situations if their parent showed symptoms of illness [38]. One girl (aged 5 years) with a dad who had diabetes and used an insulin pump said: ‘This one beeps if he has low blood sugar . . . and then he needs to get chocolate milk’ [38, p. 716].

Some adolescents emphasised the importance of being ordinary, and said that they hid their situation at home from friends by taking a ‘time out’ and entering a role as ‘ordinary youth’ [37, 50, 52]. Being ordinary is also described in the participants’ narratives in Bønnhoff and Larsen [34], who stated that they performed a ‘normal family role’, not only to hide shame due to their parent’s alcohol dependency but also to maintain the family’s self-image. However, this strategy of hiding could also be a way of avoiding shame, of showing loyalty to the PMI/PSD/PSPI, or avoiding being pitied [52, 55].

Children of parents with dementia described their attempts to provide care through joy and well-being: ‘(I) try to be strong when I visit her (mother with dementia) to smile, joke, have fun together. We dance to Elvis, eat ice cream and goodies. Have a nice time’ [42, p. 8). One way of handling the situation was to leave home at an early age to be able to have both emotional and physical distance from their ill parent and to put their own needs first [34, 42, 50, 56].

However, some children and young people as next of kin took on more care responsibilities than others as a way of maintaining their everyday life [39]. Results from the survey of Haugland et al. [39] showed that especially girls/young women, singles, those with divorced parents, immigrants, and those having financial difficulties took on more practical care responsibilities.

To maintain control in an uncertain everyday life some informants said that they took active initiatives to balance their needs against those of the families; they took responsibility for the family household, like carrying out practical tasks, caring for siblings and parents, contributing to a good atmosphere in the family, and visiting the home during the day to check on their family. In this way they tried to counteract unpredictability and insecurity [47, 55]. Some youths said they had adapted an ‘emergency alert mode’ to be prepared for the worst [42, 50].

## Discussion

This systematic review of the 23 articles has identified three main themes of importance in children as next of kin’s everyday life with PMI/PSD/PSPIs: (a) emotional ambivalence: promotive and hampering factors for wellbeing; (b) supportive and obstructive contextual dimensions of wellbeing; (c) children’s relational agency helped them create meaning. In the following discussion we will look into what the review based on data from Norway adds to the research and practice field of children as next of kin.

### Children’s everyday life in motion

In their reviews, Krattenmacher et al. [25] and Gladstone et al. [24] called for more knowledge on family relations and functioning, and our review shows that the influence of family affairs may vary depending on the relational contexts [34, 35, 37, 49, 50, 53] and the structural contexts [40, 43, 44, 47, 48].

Parental illness has an impact on the whole family and puts children’s everyday life in motion. Accordingly, illness in close relations is not only an individual concern but applies to the whole family. For the children in the reviewed studies, everyday life in motion means that their everyday life was characterised by unpredictability, ambivalence and contradictions. The children did not say that details about a parent’s medical diagnosis were the most crucial concern to keep everyday life running smoothly, but talked about the extent to which the illness had emotional and/or social consequences for them. This is in line with the cross-sectional study of Boumans and Dorant [58] from the Netherlands, showing that parents’ medical diagnoses were not the most relevant criterion for children’s experiences of everyday life.

Most of the children who participated in both the qualitative and quantitative studies described experiences of loneliness within their family and of maintaining control in their unpredictable everyday life [40, 47, 50]. However, in the qualitative study by Eide et al. [35], the adolescents reported increased family proximity even if they also described everyday life as living ‘in an earthquake zone’. Wangensteen et al. [51] described how the young people supported their parents and remained loyal even in situations in which most of the social network had withdrawn. Such a nuance in the findings illustrates that movable contexts can give rise to different degrees of proximity.

Two of the quantitative studies [40, 44] emphasise how parental illness or substance dependence put financial problems in motion and predicted negative wellbeing for children [44]. One qualitative study pointed out that adolescents put the family context in balance by taking occasional jobs to support the family financially [47]. Such social consequences of being next of kin have been given little attention in previous research.

Krattenmacher et al. [25] and Saragosa et al. [31] called for more studies focusing specifically on gender, immigration [29] and age [25] with respect to children as next of kin. In the survey of Haugland et al. [39] they identified stronger associations between girls/young women and caregiving activities, and in the study of Jeppesen et al. [41] girls reported lower health-related QoL than boys. The few studies that had a gendered perspective on children as next of kin showed that girls/young women were disadvantaged. When it comes to age, we still do not know enough about the youngest children’s experiences as next of kin, even though Hansen and Ersfjord [38] illustrated in their qualitative study of young children with a diabetic parent that children down to 5 years of age clearly expressed having a state of readiness. None of the studies included immigrants or toddlers. This is worrying because toddlers, preschoolers, and children with mother tongue different from the majority are methodologically more difficult to do research with, but are children that strongly need competent adults in everyday life.

### Children as active social actors

Gladstone et al. [24] identified a knowledge gap in descriptions of children’s practices in everyday life. In our review we found that the children were active agents in their own life [38, 40, 49, 50]. However, we found few studies on children as social actors and their use of relational agency. This is problematic because there is a growing amount of knowledge documenting that experiences in early childhood become embodied. Children want to contribute to their families, but adverse childhood experiences that exceed children’s prerequisites for dealing with them may contribute to allostatic overload and increase the child’s risk of becoming multimorbid as an adult [59].

Even with the low number of studies in this review we can claim that some of the studies [38, 40, 49, 50] intersect between health research and new childhood research [22, 57], which is an important contribution in the knowledgebase on children and childhood. These studies approaching children both as ‘beings’ and ‘becomings’, by describing children’s experiences here and now when living with PMI/PSPI, but also by looking at such structural variables as finances and school improvement, which may have impact on their adult lives. With increased school absenteeism and less participation in organised leisure activities than their peers [40], they might be in danger of having fewer resources and less energy in the struggle for social position as adults. Their agentic position may be limited. In addition, Johannesen et al. [42] reported that they left the family home at an earlier age than peers to escape from their parents’ dementia, and we do not know the consequences of starting adult life earlier than peers with healthy parents. Many of the children in the reviewed studies described themselves as relational agents through active beings and doings, but put this in their own terms appropriate for their age and maturity.

Our review has also shown that children need time outs, respite and dialogues with peers to create emotional and physical distance to their PMI/PSD/PSPI [37, 47, 49, 50]. Respite and time outs involved leisure activities with friends [40, 47], and anonymous communication with peers in an online forum for others in a similar situation [49, 50]. Revealing their situation in interaction with friends and peers was a way of normalising themselves and their everyday life. The importance of normalising themselves as ordinary children was in line with the findings in the review by Chikhradze et al. [29]. However, our review finds that balancing such normalising behaviour, and at the same time avoiding being identified as a child next of kin when they did not want to be, was hard to accomplish without support from at least one trusty predictable adult familiar with their everyday life situation.

Humour became important even when the organisation of daily activities became hard to figure out. Young people’s descriptions of their use of humour [42] as a strategy in handling dementia was an example of their use of agency as social actors. Humour is described in childhood research as an important feature of peers’ close relationships, and a key human attribute for creativity and cognitive playfulness [60]. As far as we have been able to identify, humour has not been a theme in previous reviews on children’s experiences of everyday life with PMI/PSD/PSPI. However, children and young people’s agency in the role as next of kin was not primarily about humour, but rather about loyalty to parents and working hard to maintain their role as an ordinary child [28]. They may stand up for their PMI/PSD when everybody else has let them down [51] and remain loyal [46, 51, 55], which may illustrate a high degree of solidarity in the child–parent relationship. They are committed to keeping the family rhythm going which is in line with Saragosa et al. [31] when talking about children’s practices as ‘just doing it’.

### Children’s lifebuoy: one trusty predictable adult

Järkestig-Berggren and Hanson [26] emphasised the importance of having significant adults in the life of children as next of kin, as these children have a more unpredictable and emotive everyday life than peers with healthy parents. One trusty adult may stabilise their everyday life, and then serve as a social lifebuoy as shown in Gullbrå et al. [37].

Our review has shown that children with PMI/PSD/PSPI found unpredictable changes in their parents’ behaviour and/or emotional expressions hard to handle. Such experiences were found to be independent of the parents’ medical diagnoses [35, 37, 42, 47, 50, 51, 55]. The child may then feel that she/he cannot rely on the people around them. Trust is fundamental in close relationships and is important for identity building in which trust enables an individual to behave without reservation [61]. Trust is something an individual must qualify to receive and not something that can be claimed. Unfortunately, the solution to unpredictability in parents’ behaviour, as often reported by children, is deafening silence and cover-up inside and outside the core-family [37, 55]. This was experienced as hard to accept and understand by the children involved. Wangensteen and Westby [53] found that having a significant adult, a grandparent, a teacher or health professionals, to trust and rely on without reservation was important for children with a parent with substance use disorder.

Parents are the persons in a child’s life that both legally and emotionally strive to provide care and be a trusty adult doing the best for the child. Unpredictability as a phenomenon has been reported in research on children as next of kin for years [62], and is an important topic in the reviewed articles. One hypothesis is that unpredictability may become even more frequently reported in the future as we know that people are becoming parents at an older age than before, and the number of dementia and cancer survivors is increasing. More knowledge on how to help children manage unpredictability, restore trust and maintain trust is needed for children, the extended family and professionals.

### Loneliness and silence

In their review, Dam and Hall [27] claim that information, dialogue and knowledge that can help to break the silence around the parents’ problems seem to be the most important assistance for children. Children and youth in many of our reviewed studies said they felt lonely in their family situation [46, 49, 50], insecure and disappointed [53, 55] due to the lack of information and openness in the family, social network and professionals’ network. When family members make a silent agreement, intentional or not, not to disclose the health condition of the PMI/PSD/PSPI and/or problems to the children, Chui and Yeung [63, p. 887] call this a ‘conspiracy of silence’. Such agreements, the articles reveal, could be made for different reasons and by professionals, family members or the social network. Perhaps it is too difficult to talk about, the terminology is lacking or is too hard, or one tries to protect the child from depressing information – for example, attempt to protect the child from society’s stigmatising attitudes towards some medical diagnoses or from the social consequences of the diagnoses of the PMI/PSD/PSPI.

Some of the adolescents in our review described it as easier to accept that the PMI/PSD/PSPI is unable to start a dialogue about their situation than to accept that the healthy parent and even health professionals avoid doing so [37, 55]. However, other studies have shown that nurses working with children as next of kin find it emotionally hard to talk to them about their parents’ illnesses [64] and they lack the tools to do it [65]. In Gullbrå et al. [37] the adolescents said that when they consulted their GP for various symptoms, they felt the GP failed to ask questions about their parents’ problems and their own wellbeing.

## Strengths and limitations

The strength of our review is that the research group is interdisciplinary and broadly composed of experienced researchers and clinicians within the field of mental health, substance abuse, health and welfare services, as well as researchers in childhood studies, and with skills from both qualitative and quantitative methods. This ensures relevant perspectives and questions to the material through the whole research process. In addition, as far as we have been able to identify, this is the first systematic review combining qualitative and quantitative studies, and includes children having a parent with mental illness (PMI), a parent with substance dependence (PSD) or a parent with severe physical illness/injury (PSPI) focusing on the children’s practices and experiences in their everyday life in Norway expressed by the children themselves. Children as next of kin from families with parents having different diagnoses and problems have much in common and need much of the same support, which demonstrates that children as next of kin at an aggregated level are a public health issue. Studies including Norwegian data were almost non-existent in the former reviews, which made us curious about the Norwegian knowledge contribution to the topic as Norway is one of the first countries worldwide that legalised healthcare professionals’ duty to care for children as next of kin’s needs. We presumed that Norway as a high-income country case make our findings on the children’s and youth’s descriptions of loss, sorrow, shame, guilt, meaning, agency and practical issues transferrable to other western countries, especially the Nordic countries. By including qualitative and quantitative designed studies in our systematic review, the research question has been studied from a wide range of methodological angles, and presumably strengthens the validity of our results [32].

Collaborating with a professional academic at the library when sampling was a quality-assurance procedure increasing the probability of identifying studies of relevance. However, when we know that studies are categorised according to their keywords, no procedure can guarantee that all studies were identified. All authors screened the abstracts together. On the one hand this procedure made all authors closely involved in the material and well qualified to make an opinion and participate in the solutions. On the other hand, we cannot disregard that such a procedure might have impeded discrepancies coming to the surface. We decided not to weigh the articles’ results when presenting the results section in our review. However, we followed the seven quality assurance criteria of Harden et al. [32] and because only nine [36, 37, 39–41, 43–45, 48] studies of the 23 included studies did not receive a full score on only one criterion (explicit theoretical framework), we chose to proceed in line with seven of the eight former reviews on the reviewed topic [24–31].

We claim that to include children living with parents who suffer from a variety of illnesses and dependencies is a strength of our study, but we are aware that we risk overlooking important nuances in the internal family life and thus the needs of the children. This means we still need knowledge from studies including a variety of parental problems and from studies based on the parents’ diagnoses.

## Conclusions

Children as next of kin constitute a large number of children in Norway [9] and internationally [7, 10, 12]. The children’s voices in the present review have demonstrated what they need to maintain everyday life, which is a prerequisite for offering them relevant services. At present, their developmental health and wellbeing are at stake, and a public health initiative is needed to relieve their difficult situations, both to help them in their present everyday life and to prevent psychosocial problems later.

The present review adds new knowledge supplementing previous reviews on the experiences of children and young people as next of kin:

- On the children’s relational agency, they talk about attempts to maintain their everyday life by doing practical tasks [38, 42, 43, 47, 55] and occasional jobs to support their family’s financial situation [39, 44, 47], organising fun activities with the ill parent [42, 48]. They seek respite and participation in peer activities to ‘normalise’ their everyday lives.
- On lack of information and provision of care, the children report that they lack information and provision of care for themselves and their parents. They talk about adults’ silence and lack of dialogue on their parents’ problems [34. 37, 46, 49, 50, 55].
- On significant others, they talk about the importance of having at least one trustworthy adult recognising them, and they appreciate talking with peers who have shared similar experiences as next of kin [42, 49, 50, 52].
- On ambivalent feelings, fear of heritage and dying, the review confirms previous findings [24] that children as next of kin love their parents but also feel guilt, anger, disappointment and shame. What our review adds is that children report fear of inheriting their parents’ illness or substance dependence, and some feared their parents might die [38, 47, 50, 51, 53].
- On implementation of programmes, we still lack information from children as next of kin on their experiences of the implementation of different preventive programmes as both Gladstone et al. [24] and Järkestig-Berggren and Hansen [26] call for, and there is a lack of documented good enough tools/procedures for health professionals to identify all children who are next of kin/YCs [30].
- On possible different experiences in age, gender and immigrant background, gender differences are reported in disfavour of girls [39, 45], but we lack knowledge on the experiences told by toddlers, preschoolers [38] and children with an immigrant background.

In total, a high number of children are or might become next of kin of PMI/PSD/PSPI. The present review shows that it is still highly relevant to increase the education of parents, relatives, teachers, health and social professionals, and even politicians.

## Supplemental Material

sj-docx-1-sjp-10.1177_14034948241232040 – Supplemental material for Children as next of kin’s experiences, practices, and voice in everyday life: a systematic review of studies with Norwegian data (2010–2022) Supplemental material, sj-docx-1-sjp-10.1177_14034948241232040 for Children as next of kin’s experiences, practices, and voice in everyday life: a systematic review of studies with Norwegian data (2010–2022) by Borgunn Ytterhus, Marit Hafting, Vibecke Ulvær Vallesverd, Eli Marie Wiig, Ellen Katrine Kallander and Marianne Vibeke Trondsen in Scandinavian Journal of Public Health
